# Exon-dependent transcriptional adaptation by exon-junction complex proteins Y14/RNP-4 and MAGOH/MAG-1 in *Caenorhabditis elegans*

**DOI:** 10.1371/journal.pgen.1010488

**Published:** 2022-10-31

**Authors:** Jesus Fernandez-Abascal, Lei Wang, Bianca Graziano, Christina K. Johnson, Laura Bianchi

**Affiliations:** Department Physiology and Biophysics, University of Miami Miller School of Medicine, Miami, Florida; Max-Planck-Institut fur Herz- und Lungenforschung W G Kerckhoff-Institute, GERMANY

## Abstract

Transcriptional adaptation is a powerful gene regulation mechanism that can increase genetic robustness. Transcriptional adaptation occurs when a gene is mutated and is mediated by the mutant RNA, rather than by protein feedback loops. We show here that transcriptional adaptation occurs in the *C*. *elegans clh* family of Cl^-^ channels and that it requires exon-junction complex (EJC) proteins RNP-4, MAG-1, and eiF4AIII. Depending on which exons are deleted in distinct *clh-1* alleles, different *clh* genes are regulated in an EJC-dependent manner. Our results support the idea that different transcriptional adaptation outcomes may be directed by the differential interaction of the EJC with its target mutant RNAs.

## Author summary

The expansion of molecular tools designed to introduce mutations in genes across different models has revealed that sometimes mutations do not cause any apparent phenotype. This phenomenon is called genetic robustness and it can be mediated by the mechanism of transcriptional adaptation. In transcriptional adaptation, the degradation of the mutant RNA causes the up or downregulation of genes that functionally compensate for the mutant gene. Using the genetically amenable nematode *C*. *elegans*, we show here that transcriptional adaptation depends on proteins of the Exon Junction Complex, a protein complex important for RNA stability and localization, and protein translation. Further, we show that different mutations of the same gene lead to different transcriptional adaptation outcomes and variable functional compensation. Our results bring new insights into the still poorly understood phenomenon of transcriptional adaptation.

## Introduction

Transcriptional adaptation is a form of genetic compensation in which the mutation in a gene leads to the change in the expression level and/or pattern of related genes. This phenomenon has been confirmed in zebrafish, mouse cell cultures, and more recently in *Caenorhabditis elegans* [1–4]. It has also been suggested in other model organisms, including yeast, *Arabidopsis*, *Drosophila*, and mouse [5–9]. Transcriptional adaptation does not depend on the loss of protein function but rather on mutant mRNA. At least two models of transcriptional adaptation have been proposed in which the common denominator is the presence of a premature termination codons (PTC) in the mutant mRNA. One model proposed by Ma and colleagues, is based on studies in zebrafish and it involves the interaction of the PTC containing mutant mRNA with the histone modifier COMPASS complex, leading to enhancement of histone H3 Lys4 trimethylation at the transcription start site regions of the adapting genes, which causes their upregulation [4]. This type of mechanism may co-regulate genes in operons. Another model involves the degradation of the PTC containing mRNA via a process termed nonsense-mediated decay (NMD) and the formation of small RNA species that then interact with RNA binding proteins, are transferred to the nucleus, and may regulate gene expression via interaction with histone modifiers [1,2,10].

Serobyan and colleagues recently showed for the first time that transcriptional adaptation occurs also in *C*. *elegans*. The authors showed that in this organism, transcriptional adaptation of actin and titin genes requires the function of factors involved in mRNA decay, as well as of Argonaute proteins and Dicer, which are involved in small RNA maturation and transport into the nucleus, thus supporting a model involving PTC mediated NMD [3]. Furthermore, SPK-1 and RSP-6, two serine/arginine proteins involved in mRNA binding and splicing [11,12], and homologous to components of the exon-junction complex, were recently found to also participate in transcriptional adaptation [3]. The EJC has been known to enhance NMD, via recruitment of UPF1 (an RNA decay factor called SMG-2 in *C*. *elegans*), when deposited on a mRNA containing a PTC [13]. However, other EJC proteins, including core proteins Y14 (RNP-4 in *C*. *elegans*), MAGOH1 (MAG-1 in *C*. *elegans*), and eiF4AIII (F33D11.10 in *C*. *elegans*) have not been tested for their involvement in transcriptional adaptation [14–19]. Moreover, the involvement of EJC proteins in transcriptional adaptation raises the possibility of different transcriptional adaptation outcomes based on different exon/exon junctions present in distinct mutations of the same gene [9]. The conservation of basic molecular mechanisms from *C*. *elegans* to higher organisms, urges the exploitation of this pioneering organism to better understand transcriptional adaptation, a potential modifier of disease severity that could be harnessed for treatment.

The *clh* family of Cl^-^ channels in *C*. *elegans* consists of six genes, named *clh-1* through *clh-6*, located on chromosome II (*clh-1-3* and *clh-5*), chromosome V (*clh-6*), and chromosome X (*clh-4*). *clh* genes are homologous to the mammalian CLCN genes, are expressed in various tissues, and participate in several important biological processes [20–24]. For example, CLH-1 regulates pH in the amphid sensory organ of *C*. *elegans*, mediates Cl^-^ efflux from amphid glia for GABA regulation of the mechanosensory neuron ASH, and participates in regulating the activity of sensory neurons that modulate the navigation in response to food [25–27]. Furthermore, the knock-out of *clh-1* causes wider body and abnormal alae structure, underscoring the function of this Cl^-^ channel in the hypodermal seam cells [20]. Among the six *clh* genes, *clh-1* is the only one for which three different mutants have been isolated [20,25,28], and thus, it represents a useful tool to study transcriptional adaptation.

In this study, using real time PCR, RNA interference, phenotypic measurements, quantification of the brood size, and nose touch behavioral assays, we sought to determine whether transcriptional adaptation occurs in the *clh* family and what factors might be involved. Using the three *clh-1* mutants, we report here the following findings for the *clh* family that may apply to other gene families: 1) transcriptional adaptation is allele-specific, 2) transcriptional adaptation involves the upregulation of some genes and the downregulation of others, 3) the EJC proteins RNP-4, MAG-1, and F33D11.10 (from here on referred to as eIF4AIII) are required for transcriptional adaptation, and 4) functional compensation correlates with downregulation of genes of the same family. Our study shows that different transcriptional adaptation outcomes with variable functional compensations are directed in different mutants of the same gene, adding to our understanding of this important genetic compensation mechanism.

## Results

### Transcriptional adaptation leads to different adapting gene profiles in *clh* mutant alleles

**Fig 1:**
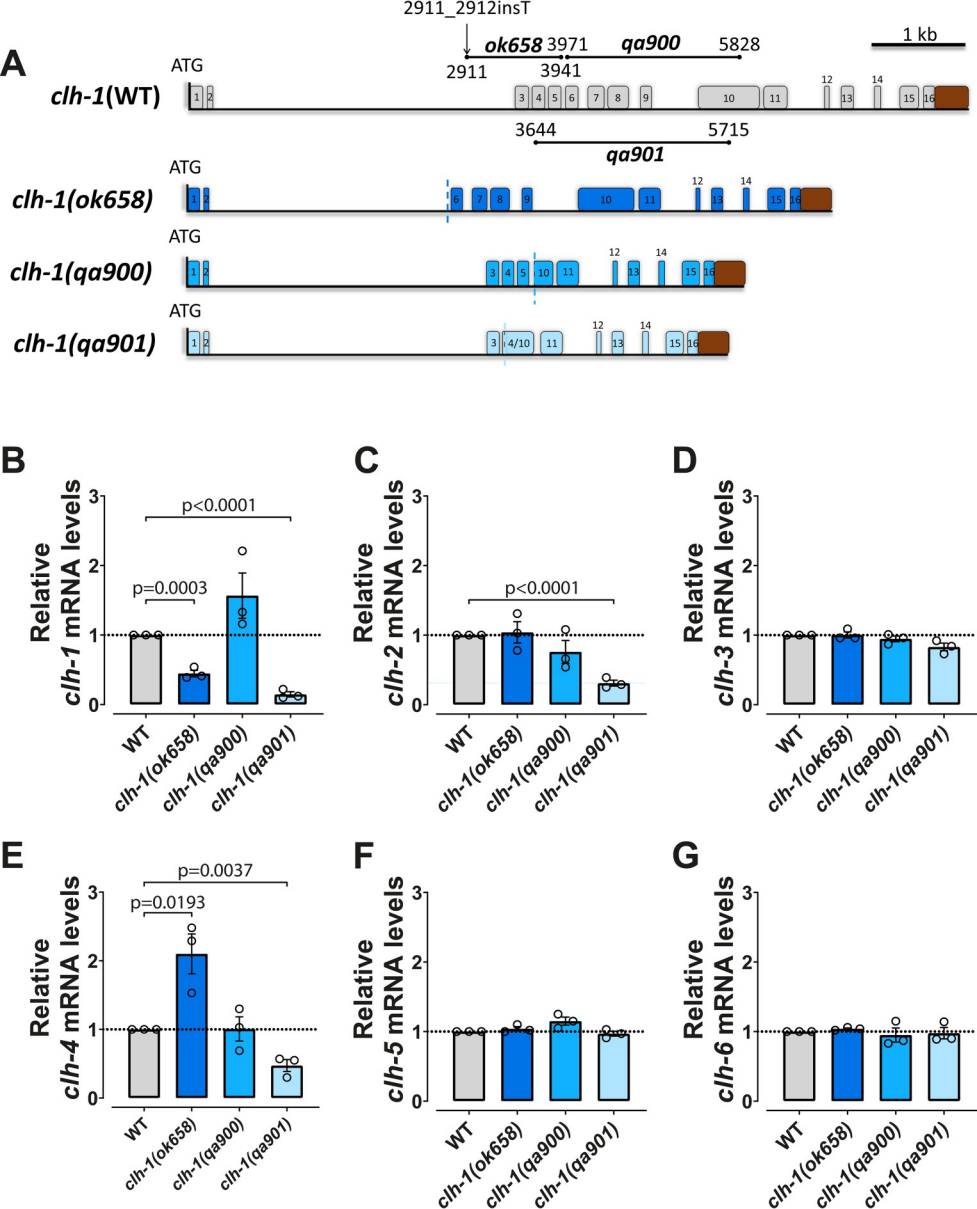
Different patterns of regulation of *clh* genes in three different *clh-1* mutants. (A) Schematic representation of the *clh-1* gene in WT and mutants. Each numbered box represents an exon, while lines are introns. The vertical line at the beginning of each gene represents the ATG start codon. The brown box represents the 3’UTR. The length and position of the deletions of each mutant and the insertion in the *ok658* mutant are also shown in the wild type gene structure. The vertical dashed lines in each of the mutants represent the deletion position. (B-G) mRNA levels of the six *clh* genes in WT and mutant alleles. Data are expressed as mean ± SEM and normalized to WT levels, that were taken as 1. The *pmp-3* mRNA levels were used as internal control. Three independent experiments with three technical replicates were performed. The horizontal dotted line in the graphs corresponds to the level in WT. All statistical differences are reported in the graphs (unpaired two-tailed t-test). Data used for this figure are reported in the S1 Table.

We first acquired three *clh-1* knock-out strains (*ok658*, *qa900*, and *qa901*) [20,25,28] and determined whether the mRNA levels for the six *clh* genes was altered in these mutants (Fig 1). The *clh-1(ok658)* mutation consists of the deletion of 1029 bp containing exons 3–5 and a thymidine insertion at position 2911_2912 [28] (Fig 1A). In this mutant, a premature stop codon (PTC) is introduced at position 313–315 of the mRNA sequence (the wild type RNA length is 2613 bp) (S1A Fig). The *clh-1(qa900)* has an in-frame deletion of 1857 bp containing exons 6–9 and part of exon 10 [20], and the *clh-1(qa901)* has a deletion of 2071 bp containing exons 4–9 and part of exon 10 [20] (Fig 1A). In *qa901* a PTC is introduced at position 511–513 of the mRNA sequence (S1A Fig).

We found that in these three mutants, the levels of *clh-1* mRNA are different (Fig 1B). While in alleles *ok658* and *qa901* the *clh-1* mRNA is reduced as compared to wild type (ratio of each *clh-1* mRNA in mutant versus wild type: 0.45 ± 0.05 and 0.15 ± 0.04, respectively), in *qa900* mutant it is on average at the same level (1.57 ± 0.33, not statistically different than wild type). These results are consistent with the NMD phenomenon, by which PTCs are recognized as signals to target RNA for degradation [29]. Indeed, the only mutant in which *clh-1* mRNA level is like wild type is *qa900*, which consists of an in-frame deletion. Consistent with the idea that transcriptional adaptation is not activated in mutants lacking a PTC, the mRNA levels of all the other *clh* genes are unaltered in *qa900* (Fig 1C–1G). On the other hand, we found that in both *ok658* and *qa901* mutants, other *clh* genes had different mRNA levels as compared to wild type animals, suggesting that transcriptional adaptation is operative in these mutants. Interestingly though, there are differences between the two mutants (Fig 1B–1G and Table 1). *clh-2* mRNA level is smaller in *qa901* but unaltered in the *ok658* mutant (Fig 1C, 0.31 ± 0.04 and 1.04 ± 0.15 versus wild type, respectively), whereas *clh-4* mRNA levels are higher in *ok658* and lower in *qa901* (Fig 1E, 2.10 ± 0.29 and 0.47 ± 0.09 versus wild type, respectively). In both mutant alleles, the expression levels of the other genes, *clh3*, *clh-5*, and *clh-6*, are like in wild type (Fig 1D, 1F–1G). To confirm these data, we performed additional qRT-PCRs using probes spanning other exon boundaries in *clh-1*, *clh-2*, and *clh-4* mRNA*s* and we obtained similar results (S1B–S1D Fig). These data show different transcriptional adaptation profiles based on the mutant *clh-1* transcript.

**Table 1:** Mutant *clh-1* and *unc-89* that cause transcriptional adaptation and their adapting genes.

Genotype	Adapted genes	Change
***clh-1 (ok658)***	*clh-4*	Upregulated
***clh-1 (qa901)***	*clh-2*	Downregulated
	*clh-4*	Downregulated
***unc-89***	*sax-3*	Upregulated

### The three *clh-1* mutants exhibit different body and brood sizes

**Fig 2:**
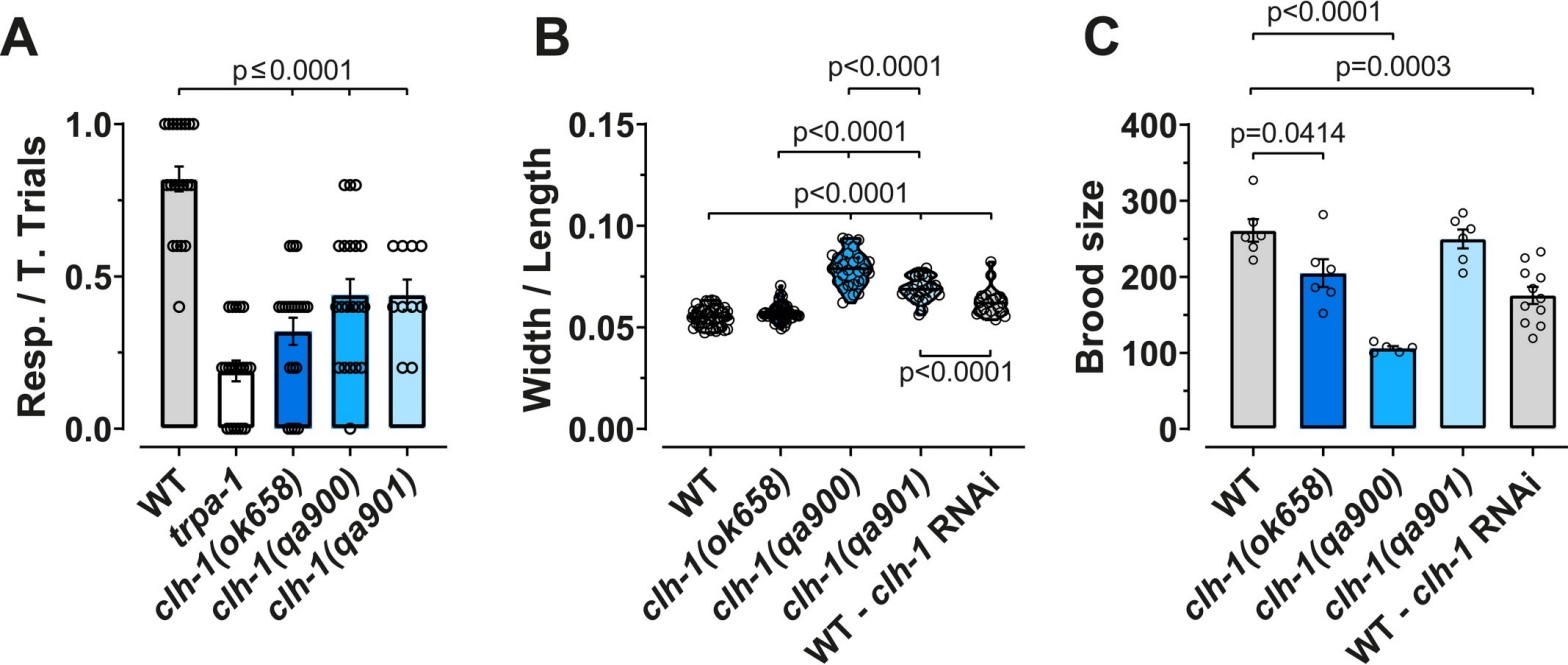
Variable phenotypic compensation in the three *clh-1* mutants. (A) Nose touch responses in the three *clh-1* mutants; wild type and *trpa-1* worms were used as positive and negative controls respectively [27,31]. Data are expressed as individual animals (open circles) and as mean ± SEM, n = 20 except *qa901* that was 10. (B) Body size ratios (width/length) of WT, *clh-1* mutants, and *clh-1* RNAi. The length of the worms was determined by measuring the distance between the tip of the nose and the end of the tail in anaesthetized animals laying straight on agarose pads. The width was determined by measuring the distance between the vulva opening and the back of the worm. Data are expressed as individual data points (open circles) and as violin plots (n = 44, 45, 27, 25, and 20 respectively). (C) Brood size of WT, *clh-1* mutants, and *clh-1* RNAi. The columns represent the average brood size of each genotype, and the open circles represent individual worms (n = 6, 6, 5, 6, and 11 respectively). Data represent mean ± SEM and were obtained by counting the number of adults found in plates where individual worms were grown for 24 hours over 5 consecutive days. Statistical analysis was by one-way ANOVA followed by Tukey’s. Statistical differences are shown in the graphs as p values. Data used for this figure are reported in the S1 Table.

Transcriptional adaptation is thought to provide functional compensation via change in expression of related genes (reviewed in [30]). Thus, we wondered whether *qa900*, the mutant allele in which there is no change in expression of the other *clh* genes, displayed a phenotype different from *ok658* and *qa901*. Moreover, we asked whether *ok658* and *qa901*, which display different mRNA profiles for *clh-2* and *clh-4*, differed in their phenotypes. *clh-1(ok658)* mutants are nose touch insensitive, and *clh-1(qa900)* and *clh-1(qa901)* have wider bodies [20,27]. We thus compared the nose touch phenotype and body size across all three mutants (Fig 2A and 2B). In addition, we compared brood size since we noticed that *clh-1(qa900)* produced significantly fewer progenies (Fig 2C). When we compared nose touch avoidance across the three mutants, we found no statistical differences (avoidance index was 0.32 ± 0.045, 0.44 ± 0.052, and 0.44 ± 0.05 for *ok658*, *qa900*, and *qa901*, respectively) (Fig 2A) [27]. Thus, the nose touch insensitive phenotype is shared by the three *clh-1* mutants, suggesting that this phenotype is not functionally compensated.

On the other hand, we found differences in the other two phenotypes. For body size, we found that while *ok658* mutants are similar to wild type in width and length (width, WT = 57.88 ± 0.66 μm, *ok658* = 59.31 ± 0.67 μm and length, WT = 1050 ± 10.16 μm, *ok658* = 1036 ± 11.09 μm, respectively), *qa900* and *qa901* mutants are wider and shorter (width, *qa900* = 68.31 ± 1.43 μm, *qa901* = 65.74 ± 1.36 μm and length, *qa900* = 869.9 ± 11.41 μm, *qa901* = 952.4 ± 12.95 μm, respectively), as it was previously reported (S2A Fig) [20]. The difference in body proportions is particularly evident in Fig 2B, where we plotted the ratio between width and length. These data show that *qa900* mutant has the most severe phenotype having widest and shortest body. Thus, these results suggest that changes in expression of other *clh* genes in *clh-1(ok658)* and *clh-1(qa901)* mutants may result in compensation of the wider and shorter body phenotype.

When we analyzed the brood size in the three *clh-1* mutants, we once again found that *qa900* was the mutant with the most severe phenotype having the smallest brood size among the three mutants (brood size in WT, *ok658*, *qa900*, and *qa901* was 249.8 ± 12.39, 204.8 ± 18.21, 106.2 ± 2.7, and 261 ± 14.9, respectively) (Fig 2C). Taken together, these data suggest that the body size and the brood size may be phenotypes that are transcriptionally compensated in both *ok658* and *qa901* mutants.

### The specificity of transcriptional adaptation in the *clh-1* mutants

**Fig 3:**
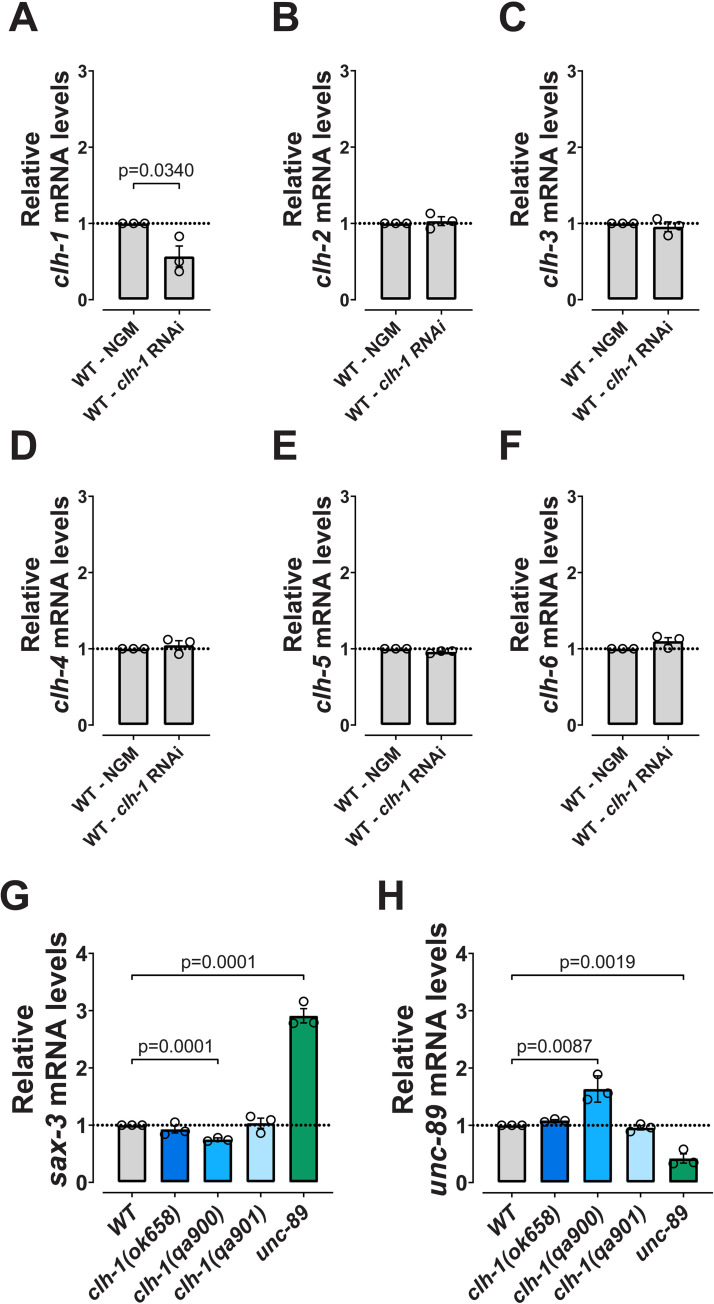
Transcriptional adaptation is dependent on mutant RNA and is gene-family specific. (A-F) mRNA levels of the six *clh* genes in wild type animals treated with *clh-1* RNAi compared to control growth conditions. (G) mRNA levels of *sax-3* in *clh-1* and *unc-89* mutants. (H) mRNA levels of *unc-89* in *clh-1* and *unc-89* mutants. Data are expressed as mean ± SEM and normalized to WT levels, that were taken as 1. The *pmp-3* mRNA levels were used as internal control. Three independent experiments with three technical replicates were performed. The horizontal dotted line corresponds to the level in control conditions (A-F) and in WT (G and H). All statistical differences are reported in the graphs (unpaired two-tailed t-test). Data used for this figure are reported in the S1 Table.

Transcriptional adaptation is induced by mutations in the genome but not by the knockdown of a gene [1]. To gather further support that the changes in *clh-2* and *clh-4* mRNA levels seen in *ok658* and *qa901* mutants might be due to transcriptional adaptation, we performed *clh-1* knockdown experiments. While we confirmed knockdown of *clh-1* (Fig 3A), we found no differences in the levels of mRNA of the other *clh* genes (Fig 3A–3F). These data lend further support to the idea that the different mRNA levels for *clh-2* and *clh-4* observed in *ok658 and qa901* are due to transcriptional adaptation. Next, we analyzed body and brood size in *clh-1* RNAi worms and found that these phenotypes were significantly different than wild type (Fig 2B and 2C). Parenthetically, *clh-1* RNAi also causes nose touch insensitivity [27]. These data further support the idea that the wider and short body, and the smaller brood size are due to uncompensated loss of *clh-1* function in *C*. *elegans*.

To test whether changes in *clh-2* and *clh-4* gene expression in *clh-1* mutants were specific, we analyzed the transcript of titin-related gene *sax-3* in the three mutants (Fig 3G). *sax-3* mRNA undergoes transcriptional adaptation in mutants of the titin gene *unc-89*, so we added *unc-89* mutant as positive control [3]. As previously reported by Serobyan and colleagues, in *unc-89* mutants the adapting gene *sax-3* is upregulated (Fig 3G and Table 1), while the mRNA level of *unc-89* transcript itself is lower than in WT, consistent with the NMD process (Fig 3H) [3]. We found that neither *sax-3* or *unc-89* mRNA levels were altered in *ok658* and *qa901* mutant alleles (Fig 3G and 3H). Interestingly, we found slight downregulation of *sax-3* and slight upregulation of *unc-89* in *qa900* mutant, that might be related to the severely altered body size and proportions in this mutant, given that *unc-89* and *sax-3* encode for titin-related genes. Taken together, these data support that changes in the expression of *clh-2* and *clh-4* seen in *ok658* and *qa901* mutants are not stochastic.

### The transcriptional adaptation of *clh-2* requires RNA biogenesis factor ERGO-1

To gather further support for transcriptional adaptation, we looked at the similarity between *clh-1* and the other *clh* genes. Indeed, transcriptional adaptation has been reported to involve more frequently similar genes, even though non-similar genes have been also shown to undergo up or down regulation in PTC-bearing mutants [2]. Using blastn and a word size of 20, we found significant similarity between *clh-1* and *clh-2*, *clh-3*, *clh-4*, and *clh-5*. More specifically, we found that *clh-2* contains a continuous stretch of 20 bp that is 100% identical to a stretch of nucleotides in *clh-1* exon 13. In addition, *clh-2* shares 68% to 85% homology with *clh-1* in stretches of nucleotides varying in length between 32 and 228 bp across 785 bp total. We also found significant homology with *clh-3* (70% identity across 320 bp), *clh-4* (66% identity across 113 bp), and *clh-5* (88% identity across 49 bp). These similarities further support the idea that the differences seen in *clh-2* and *clh-4* mRNA levels in *clh-1(ok658)* and *clh-1(qa901)* might be due to transcriptional adaptation. To gather experimental evidence for this conclusion, we quantified the pre-mRNA levels of *clh-2* and *clh-4* and of *clh-4* in the *qa901* and *ok658* mutants, respectively. We found that the pre-mRNA levels of *clh-2* and *clh-4* are smaller than in WT in the *qa901* mutant, and that the pre-mRNA level of *clh-4* is higher than in WT in the *ok658* mutant (S1E–S1F Fig). Thus, the changes in steady state RNA levels of *clh-2* and *clh-4* seen in *qa901* and *ok658* (Figs 1C and 1E and S1C-) correspond to changes in transcription of these genes, supporting the idea that they are the result of transcriptional adaptation.

Next we asked whether ERGO-1, a protein involved in small RNA biogenesis [32–35], and required for transcriptional adaptation in *C*. *elegans* titin and actin families [3], was required for changes in *clh-2* and *clh-4* mRNA levels in *clh-1* mutants. First, consistent with the idea that ERGO-1 regulates transcriptional adaptation downstream of mRNA decay, we found that the mutant *clh-1* mRNA levels were still downregulated in *ergo-1* RNAi (S1G Fig), whose effectiveness was confirmed by qRT-PCR (S1J Fig) [3]. Second, we found different outcomes for *clh-2* and *clh-4* mRNAs in *ergo-1* RNAi. While the mRNA levels of *clh-2*, which were downregulated in *qa901* (Fig 1G), were now upregulated (S1H Fig, upregulation of *clh-2* in *ergo-1* RNAi was also seen with *clh-2* probe spanning 11–12 exons: 2.247 ± 0.29 relative to WT), the mRNA levels of *clh-4* for *ok658* and *qa901* were unchanged as compared to control conditions (S1I Fig). These results support the requirement for small RNA biogenesis for *clh-2* downregulation in *qa901*, but not for the changes in *clh-4* mRNA levels in *ok658* and *qa901*. Intriguingly, *clh-2* is the only *clh* gene in which a 20 bp sequence sharing 100% identity with *clh-1* is found. Taken together, these results suggest that different transcriptional adaptation mechanisms may be operative in the same mutant to target different adapting genes.

### Transcriptional adaptation of *clh* genes requires the Exon Junction Complex

Serobyan and colleagues reported a list of proteins involved in transcriptional adaptation, including splicing factors *spk-1* and *rsp-6* [3]. However, the core components of the EJC Y14/RNP-4, MAGOH/MAG-1, and eIF4AIII were not included in their study [14,15,17–19,36]. The EJC is involved in transcriptional adaptation via its role in NMD. EJCs are deposited on the mRNA during splicing in the nucleus, remain on mRNAs even after transport to the cytosol, and are then removed from the mRNA by the ribosome during the pioneer round of translation. If a PTC is present upstream of an EJC, then this is not dislodged from the mRNA during translation leading to the recruitment of RNA decay factor UPF1 (*smg-2* in *C*. *elegans*) that degrades the RNA [13].

**Fig 4:**
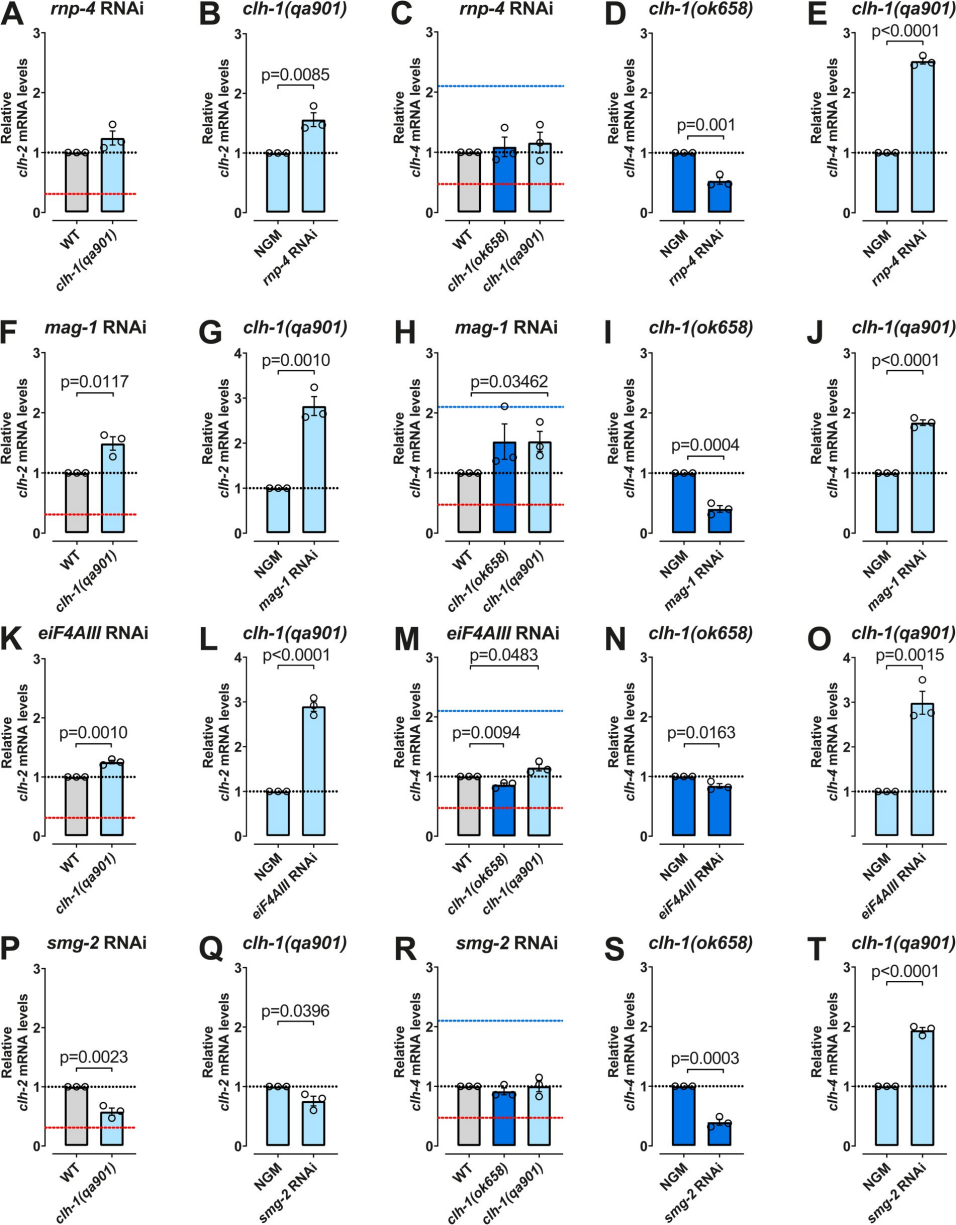
Exon-junction proteins and the non-sense mediated decay protein SMG-2 mediate transcriptional adaptation of *clh* and titin genes. (A-E) mRNA levels of *clh-2* and *clh-4* in *clh-1* mutant alleles upon knockdown of *rnp-4*. (F-J) mRNA levels of *clh-2* and *clh-4* in *clh-1* mutant alleles upon knockdown of *mag-1*. (K-O) mRNA levels of *clh-2* and *clh-4* in *clh-1* mutant alleles upon knockdown of eiF4AIII (P-T) mRNA levels of *clh-2* and *clh-4* in *clh-1* mutant alleles upon knockdown of *smg-2*. Data are expressed as mean ± SEM and normalized to WT levels or NGM condition, that were taken as 1 (black dotted line). Three independent experiments with three technical replicates were performed, and *pmp-3* mRNA levels were used as internal control. Dashed lines correspond to mRNA levels of *qa901* in NGM (red, from Fig 1C and 1E) and mRNA levels of *ok658* in NGM (blue, from Fig 1E). The horizontal black dotted line corresponds to the levels in control conditions or WT, as indicated. All statistical differences are reported in the graphs (unpaired two-tailed t-test). Data used for this figure are reported in the S1 Table.

To experimentally determine the potential role of EJC proteins in transcriptional adaptation in *clh-1* mutants, we performed knockdown by RNAi feeding in wild type and *clh-1* mutants, as well as in the *unc-89* mutant as a control (Figs 4, S3 and S4 and Table 2). When we analyzed the mRNA levels of the adapting genes *clh-2* and *clh-4* in *clh-1* mutants, and of *sax-3* in *unc-89* mutant treated with *rnp-4* RNAi, we found significant reduction of transcriptional adaptation (Figs 4A–4E, S3G and S3H). Thus, the *clh-2* mRNA levels were no longer downregulated in *qa901* worms but were like the levels seen in wild type (Fig 4A) and, therefore, higher than the levels seen in *qa901* grown under control conditions (Fig 4B). The *clh-4* mRNA levels were no longer up- and downregulated in *ok658* and *qa901* worms, respectively (Fig 4C), thus they were lower and higher than the levels observed in these mutants grown in control conditions (Fig 4D and 4E, respectively), and the *sax-3* mRNA levels were no longer upregulated in *unc-89* worms (S3G Fig). We also found that the *clh* genes that do not undergo transcriptional adaptation in both *clh-1* mutants remained unaltered when *rnp-4* was knocked down (S3A–S3C Fig). Interestingly, the mRNA levels of the mutant *clh-1* were still lower than the wild type (S3D Fig). However, comparison with the control conditions reveled that the mutant *clh-1* RNA is not as low in *qa901*, suggesting reduced degradation in this mutant (S3E Fig), a phenomenon not observed in *ok658*, despite similar *rnp-4* knockdown efficiency in the two mutants (S3F Fig).

To determine whether the effects of *rnp-4* RNAi were specific, we knocked down the gene *ZC155*.*4* which encodes an ortholog of human glycerophosphodiester phosphodiesterase 1 (GDE1). The gene product of *ZC155*.*4* is predicted to participate in lipid metabolic processes and is not expected to be involved in transcriptional adaptation or RNA processing. We found that knock down of *ZC155*.*4* had no effect on the mRNA levels of *clh-1*, *clh-2*, and *clh-4* in *clh-1(ok658)* and *clh-1(qa901)* mutants (S3I–S3K Fig), supporting the specificity of the effects seen in *rnp-4* RNAi.

The RNAi of the other EJC component, MAG-1 caused also substantial decrease of transcriptional adaptation in both the *clh* and titin families (Figs 4F–4J and S4D). Thus, *clh-2* and *clh-4* mRNA levels were no longer downregulated in *qa901* (Fig 4F, 4G, 4H and 4J), the upregulation of *clh-4* in *ok658* worms was significantly reduced (Fig 4H and 4I), and the upregulation of *sax-3* in *unc-89* mutants was absent (S4D Fig). As seen for *rnp-4* RNAi, *mag-1* RNAi did not affect the downregulation of *clh-1* transcript in *ok658* mutant but reduced the downregulation of *clh-1* in *qa901*, suggesting reduced degradation of the *clh-1* transcript in this mutant (S4A and S4B Fig). In the case of *mag-1* RNAi too, knockdown shows the same effectiveness in the two *clh-1* mutants (S4B Fig). Finally, we tested the involvement of the core component of the EJC eiF4AIII and obtained results similar to the ones obtained with RNAi of *rnp-4* and *mag-1* (Figs 4K–4O and S4E–S4G). Taken together, these findings are consistent with the idea that the process of NMD is EJC-dependent in *C*. *elegans*. Although they also reveal that, at least in the case of *ok658* mutant, knockdown of components of the EJC does not lead to changes in degradation of the mutant RNA, suggesting an EJC-independent NMD mechanism in this *clh-1* mutant [37,38]. Thus in *C*. *elegans* EJC-dependent and EJC-independent NMD appear to exist side by side [39].

### The UPF1 homolog SMG-2 and transcriptional adaptation of *clh* genes

Serobyan and colleagues reported that *smg-2* and *smg-4* genes are required for transcriptional adaptation of *unc-89* alleles, whereas *smg-6* is required for the transcriptional adaptation in *act-5* [3]. SMG-2, SMG-4, and SMG-6 are all RNA decay factors that have been implicated in transcriptional adaptation in zebrafish embryos and mouse cell lines, in addition to *C*. *elegans* [2,4]. We thus wondered whether transcriptional adaptation in *clh-1* mutants required SMG-2, the *C*. *elegans* ortholog of the ATP-dependent RNA helicase upstream frameshift 1(UPF1*)* [40]. We found that the knockdown of *smg-2*, partially blocked the transcriptional adaptation of the adapting gene *clh-2* in *qa901* worms (Fig 4P and 4Q) and fully blocked changes in the *clh-4* transcript in *ok658* and *qa901* worms (Fig 4R–4T). As reported by Serobyan and colleagues, *smg-2* knockdown blocked the transcriptional adaptation of *sax-3* (S4K Fig) and the decay of *unc-89* mRNA in *unc-89* worms (S4L Fig) [3]. Furthermore, a block of decay of the *clh-1* transcript was observed in *smg-2* RNAi (S4H and S4I Fig), and the efficiency of the RNAi treatment was confirmed by qRT-PCR (S4J Fig), thus confirming that mutant *clh-1* transcripts are degraded via the NMD pathway. To conclude, the RNA decay factor *smg-2* is required for degradation of the mutant *clh-1* transcripts and for the changes in *clh-2* and *clh-4* mRNA levels seen in *clh-1(ok658)* and *clh-1(qa901)* mutants.

### RNP-4 and MAG-1 are not required in development for transcriptional adaptation

In the knockdown experiments we performed, we grew worms from egg to adult on RNAi plates [41]. To determine whether RNP-4 and MAG-1 are required in development for transcriptional adaptation, we repeated knockdown experiments by growing worms on RNAi plates from the last larval stage L4 to adulthood (24 hours) (S5A–S5L Fig). Under these conditions, we obtained results that were overall similar to the results obtained from animals that were reared from egg to adult on the RNAi plates, albeit the block of *clh-4* transcriptional adaptation appeared weaker (compare S5D–S5F and 5J–5L Fig with Fig 4C–4E and 4H–4J). Similarly, *smg-2* RNAi in late larvae/young adults reduced transcriptional adaptation, though not to the same extent as in experiments in which animals were reared on RNAi plates from egg to adulthood (S5M–S5N Fig). These results support the idea that RNP-4, MAG-1, and SMG-2 are not essential during development for the transcriptional adaptation observed in the *clh* family.

**Table 2:** Summary of the effects of knocking down control gene *ZC155*.*4*, *rnp-4*, *mag-1*, eiF4AIII, and *smg-2* on transcriptional adaptation. “Yes” indicates that the gene is either up or down-regulated, “no” indicates no change in transcript level as compared to wild type, and “reduced” indicates that the indicated genes were up or down-regulated but not to the same extent as in control conditions.

RNAi gene	Function	Genotype / Adapted Gene	Transcriptional adaptation
***-***	**-**	*clh-1 (ok658)* / *clh-4*	yes
		*clh-1 (qa901)* / *clh-2*	yes
		*clh-1 (qa901)* / *clh-4*	yes
		*unc-89* / *sax-3*	yes
***ZC155*.*4*** (control)	**glycerophosphodiester** **phosphodiesterase 1**	*clh-1 (ok658)* / *clh-4*	yes
		*clh-1 (qa901)* / *clh-2*	yes
		*clh-1 (qa901)* / *clh-4*	yes
***rnp-4***	**mRNA splicing (EJC)**	*clh-1 (ok658)* / *clh-4*	no
		*clh-1 (qa901)* / *clh-2*	no
		*clh-1 (qa901)* / *clh-4*	no
		*unc-89* / *sax-3*	no
***mag-1***	**mRNA splicing (EJC)**	*clh-1 (ok658)* / *clh-4*	no
		*clh-1 (qa901)* / *clh-2*	reduced
		*clh-1 (qa901)* / *clh-4*	reduced
		*unc-89* / *sax-3*	no
**eiF4AIII**	**mRNA splicing (EJC)**	*clh-1 (ok658)* / *clh-4*	reduced
		*clh-1 (qa901)* / *clh-2*	reduced
		*clh-1 (qa901)* / *clh-4*	reduced
		*unc-89* / *sax-3*	Not tested
***smg-2***	**mRNA decay and processing (NMD)**	*clh-1 (ok658)* / *clh-4*	no
		*clh-1 (qa901)* / *clh-2*	yes
		*clh-1 (qa901)* / *clh-4*	no
		*unc-89* / *sax-3*	no

### Effects of loss of transcriptional adaptation on the phenotypes of *clh-1* mutants

The three *clh-1* mutants we analyzed here have similar nose touch avoidance phenotype, but different brood size and body’s width/length phenotypes (Fig 2). Interestingly, *clh-1(qa900)* which does not contain a PTC and in which we did not observe any change in the levels of the other *clh* genes’ transcripts, displays the most severe phenotypes having the largest width/length ratio and the smallest brood size. We thus wondered whether knockdown of the factors we found to be involved in transcriptional adaptation of the *clh* family in mutants *clh-1(ok658)* and *clh-1(qa901)* would exacerbate the phenotypes in these mutants and render them more similar to *clh-1(qa900)*. Thus, we analyzed body’s size in *rnp-4*, *mag-1*, and *smg-2* knockdown animals and brood size in *rnp-4* and *smg-2* knockdown. The extremely low number of progenies in all strains treated with eiF4III RNAi prevented meaningful analysis of brood size under these conditions (S2I Fig).

**Fig 5:**
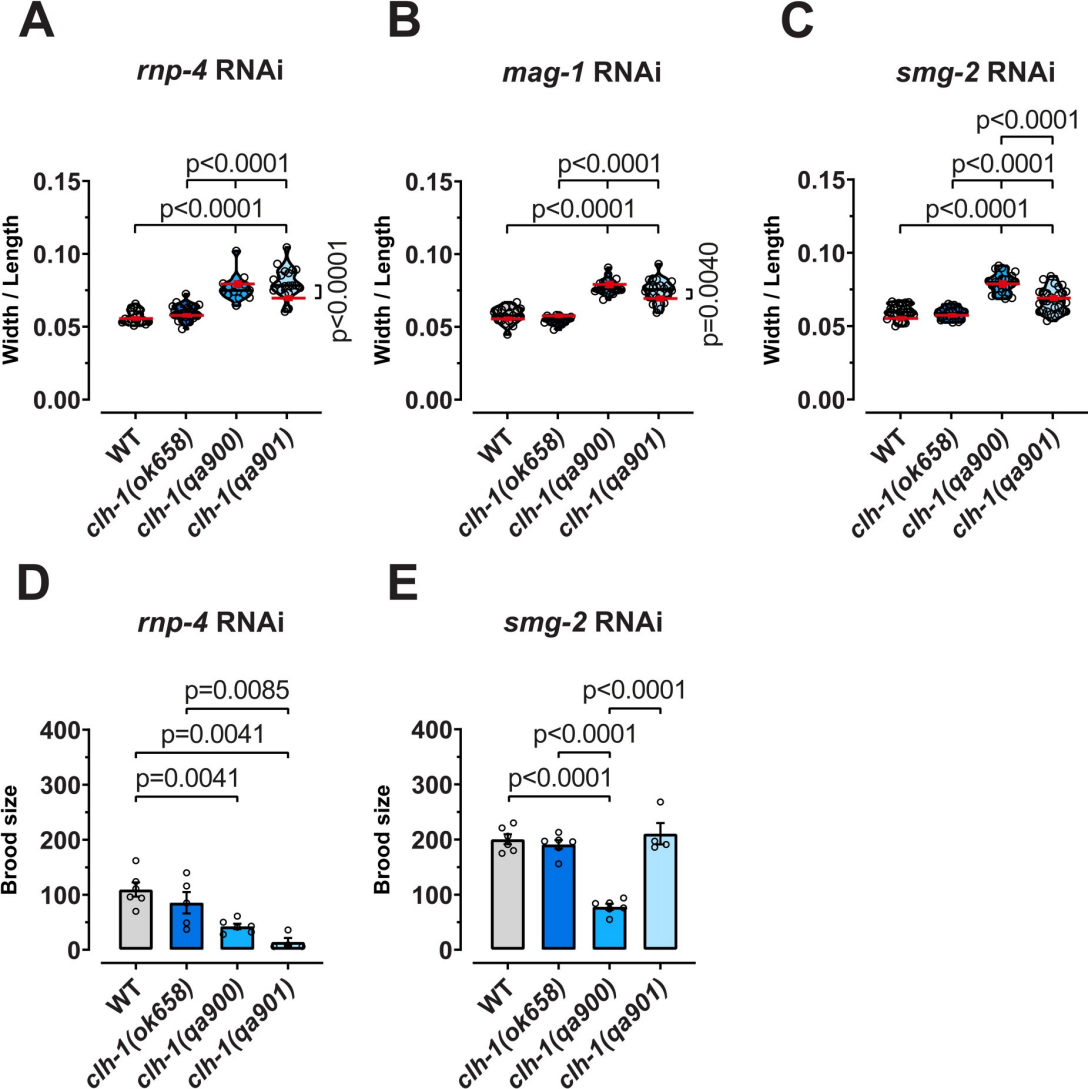
The EJC proteins RNP-4 and MAG-1 mediate functional compensation in *clh* mutants. Body size ratios (width/length) of WT and *clh-1* mutants of *rnp-4* (A), *mag-1* (B), and *smg-2* (C) RNAi treated worms. Data are expressed as individual data points (open circles) and as violin plots (n = 19, 26, 14, and 20, respectively for *rnp-4* RNAi; n = 19, 17, 21, and 22, respectively for *mag-1* RNAi; and n = 26, 21, 24, and 28, respectively for *smg-2* RNAi). The red lines are the mean of each strain in control conditions reported in Fig 2B and shown here for comparison. Brood size of WT and *clh-1* mutants following *rnp-4* (D) and *smg-2* (E) RNAi treatments. The columns represent the average brood size of each genotype, and the open circles represent individual worms (n = 6, 6, 6, and 4, respectively for *rnp-4* RNAi; and n = 6, 6, 6, and 4, respectively for *smg-2* RNAi). Data represent mean ± SEM. Statistical analysis was by one-way ANOVA followed by Tukey’s. Statistical differences are shown in the graphs as p values. Data used for this figure are reported in the S1 Table.

We found that knockdown of *rnp-4* and *mag-1* exacerbated the width/length ratio in *qa901* worms, while it did not have any effect in *ok658* (Figs 5A, 5B and S2C–S2F). Interestingly, *smg-2* knockdown, which reduced transcriptional adaptation in *ok658* and *qa901* mutants, but not as effectively as *rnp-4* and *mag-1* knockdown, did not have any effect on the body size of any of the mutants (Figs 5C, S5G and S5H). The brood size was on average decreased in all the strains in *rnp-4* knockdown, as previously reported [19]. However, the effect on the brood size of *rnp-4* knockdown was most evident in *qa901* mutant which produced a number of progenies as low as *qa900* mutant (Fig 5D). Knockdown of *smg-2* did not have any effect on the brood size either despite leading to at least partial block of transcriptional adaptation as mentioned above (Fig 5E). The results with *smg-2* RNAi suggest that the level of transcriptional adaption under these conditions is still sufficient to induce functional compensation. These results support the idea that in *qa901* mutant, knockdown of *rnp-4* and *mag-1* causes the worsening of the phenotypes, suggesting that in this mutant transcriptional adaptation leads to functional compensation. It is interesting to note that in *qa901*, both *clh-2* and *clh-4* genes are downregulated. On the contrary, even though we observe block of upregulation of *clh-4* in *ok658* mutant treated with *rnp-4* and *mag-1* RNAi, the phenotypes remain unaffected, suggesting that other genes compensate the phenotypes in *ok658*, perhaps in an *rnp-4* and *mag-1* independent manner.

## Discussion

With the expansion of genetic tools that engineer mutations in genes, there is a growing interest in understanding mechanisms of genetic compensation. Indeed, across species, genetic mutations often do not result in any apparent phenotype. *C*. *elegans* is a genetically amenable organism that can be used for rapidly advancing our understanding of the mechanisms of genetic compensation, including transcriptional adaptation.

The work that we present here adds to our understanding of transcriptional adaptation in *C*. *elegans*, so far described only in another manuscript [3]. We report here that the EJC proteins RNP-4, MAG-1, and eiF4AIII are needed for transcriptional adaptation in the *clh* and titin families and show that the transcriptional adaptation outcome of the adapting genes depends on the specific PTC-bearing mutant alleles. More specifically, transcriptional adaptation in the *clh* family can result in the downregulation or the upregulation of the adapting genes, it is, at least in part, dependent on the RNA biogenesis factor ERGO-1 [3], while the RNA decay factor SMG-2 appears commonly required, at least for transcriptional adaptation in the 2 *clh-1* mutants we analyzed [13,33–35].

Our data support that the introduction of a premature stop codon (PTC) is a key factor in promoting transcriptional adaptation in *C*. *elegans*. Indeed, we show that *qa900* mutant, consisting in an *in-frame* deletion (Figs 1A and S1), does not lead to change in the expression of other *clh* genes. On the contrary, *ok658* and *qa901* alleles introduce PTC and lead to changes in expression level of *clh-2* and *clh-4* (Fig 1), in line with what was shown by Serobyan and colleagues for the actin and titin families in *C*. *elegans* [3]. Our *smg-2* RNAi data support the idea that the PTC-bearing mRNAs undergo degradation via the NMD mechanism [42] and thus, that the lower levels of mutant *clh-1* detected in these mutants are not likely due to reduced transcription of *clh-1*. Interestingly though, this is as far as the commonality of transcription adaptation mechanism between the two PTC-bearing *clh-1* mutants goes.

Our analysis of the requirement for EJC proteins and for RNA biogenesis factor ERGO-1 for transcriptional adaptation in *ok658* and *qa901* reveals differences between the two mutants. While changes in *clh-2* and *clh-4* expression levels in both *ok658* and *qa901* mutants are blocked when EJC proteins are knocked down (Table 2), the outcome of this treatment on the PTC-bearing *clh-1* mRNA level is different. While in *ok658* it is still degraded, in *qa901* it is not (S3E, S4B and S4F Figs). This result suggests that in *ok658* the EJC complex is required for transcriptional adaptation (perhaps indirectly via other genes) [2], but it is not required for NMD of the mutant *clh-1* mRNA, consistent with an EJC-independent NMD in this mutant as previously described in *C*. *elegans* and in other systems [37,39,43,44]. This model would still be consistent with the fact that *smg-2* RNAi reduces transcriptional adaptation in both *ok658* and *qa901* mutants, given that at least in human cell lines UPF1, the mammalian homolog of SMG-2, is needed for both EJC-dependent and EJC-independent NMD [39]. Future studies in which the entire transcriptome is compared between *ok658* and *qa901* mutants may be needed to shed more light on the difference between mechanisms of transcriptional adaptation in these mutants.

Importantly, in *qa901*, where we find requirement for ERGO-1, we also observe worsening of the phenotype when EJC proteins are knocked down, consistent with the idea that in this mutant transcriptional adaptation leads to at least partial functional compensation. Indeed, under *rnp-4* and *mag-1* RNAi conditions, *qa901* body and brood size are similar to *qa900* mutant. Interestingly though, in this mutant both *clh-2* and *clh-4* mRNA are downregulated. While the study of transcriptional adaptation has been focusing primarily on understanding how adapted genes become upregulated, the downregulation of genes has been observed before [2,3]. For example, in *act-5(dt2019)* mutants, while *act-3* becomes upregulated, *act-4* becomes downregulated [3]. On a larger scale, El-Brolosy and colleagues reported the downregulation, in addition to the upregulation, of hundreds of genes in three mouse knock-out cell lines [2]. One attractive model is that small RNAs produced by the degradation of mutant *clh-1* directly silence *clh-2* and *clh-4* mRNA [45]. Alternatively, small RNA, once bound to RNA binding proteins, may act on the promoter regions of these genes (reviewed in [46]). Intriguingly, *clh-1* shares some identity with *clh-4*, and even more so, with *clh-2* (100% identity over a stretch of 20 nucleotides). Our data also show that the downregulation of *clh-2* and/or of *clh-4* lead to functional compensation. Although the involvement of other genes cannot be excluded, how might the downregulation of a gene lead to functional compensation? This is not clear, but effects mediated by antisense transcripts or the proteins themselves, especially if the proteins have opposite effects on cellular physiology, can be envisioned [2].

In neither *ok658* nor *qa901* the nose touch avoidance phenotype is compensated. Similarly, mutations in the *C*. *elegans* Na^+^/K^+^-ATPase α-subunits *eat-6* and *catp-1* cause upregulation of the homologous gene *catp-2*. However, the upregulation of *catp-2* does not compensate for the nose touch insensitive phenotype of *eat-6* and *catp-1* mutants [47]. Other examples of failed functional compensation can be found in zebrafish. For example, *vegfaa* (Vascular endothelial growth factor A) mutants, there is an upregulation of the related gene *vegfab*, but these animals still show vascular hypoplasia [48]. Failed functional compensation in transcriptional adaptation may be associated with phenotypes, such as nose touch avoidance or vascularization, that are still compatible with survival and reproduction.

Finally, we must caution on the fact that in our work we have not analyzed a full locus deletion of *clh-1*; instead, we have compared our results obtained in PTC-bearing *ok658* and *qa901* mutants with the in-frame deletion *qa900* where *clh-1* RNA is still present. On the contrary Serobyan and colleagues using an RNA-less *unc-89* mutant were able to show that transcriptional adaptation indeed requires the presence of the mutant mRNA [3]. Thus, we cannot exclude the possibility that other mechanisms such as loss of CLH-1 function are at play here.

In summary, we have shown here that the EJC plays a key role in transcriptional adaptation in *C*. *elegans*. Furthermore, we report that transcriptional adaptation can lead to either the up or down regulation of related genes, depending on the mutant allele, and that functional compensation is variable not only depending on the mutation but also depending on the phenotype. Finally, we have confirmed that transcriptional adaptation requires the presence of PTC-bearing mRNA. The data presented here urge consideration of this genetic mechanism of compensation whenever working with *C*. *elegans* knock-out strains. The good practice of the worm community of analyzing knock-out, knockdown, and rescue strains must be maintained to safeguard from misinterpretation of phenotypes. Furthermore, the genetic amenability of *C*. *elegans* makes it an excellent model to advance the study the molecular underpinnings of this important, yet still poorly understood, phenomenon.

## Materials and methods

### Lead contact

Further information and requests for resources and reagents should be directed to and will be fulfilled by the Lead Contact, Laura Bianchi (lbianchi@med.miami.edu).

### *C*. *elegans* growth and maintenance

Experiments were performed using healthy 1 day old adult hermaphrodites. Nematodes were grown on standard grow medium (NGM) seeded with *Escherichia coli* (OP50 strain) and kept at 20°C. N2 Bristol was the wild type strain.

### C. elegans strains

N2 strain from CGC was used as wild type [49]. The *clh-1* mutants, strain RB1052 *trpa-1(ok999)*, and strain VC40193 *unc-89(gk509355)* were also purchased from CGC [20,28,50]. Strains XA900 and XA901 were originally outcrossed at least 4 times [20]. Strain BLC588 *clh-1(ok658)* was obtained after outcrossing 3 times the RB833 strain. The *unc-89* strain used in this study (BLC532) was obtained after outcrossing 3 times the VC40193 strain. The full list of the strains used in this study is reported in Table 3.

**Table 3:** Nematode strains used in this study.

Genotype	Strain Name	Reference
**Wild type**	N2	CGC [49]
***trpa-1 (ok999)***	RB1052	CGC [28]
***clh-1 (ok658)***	RB833	CGC [28]
***clh-1 (ok658)***	BLC588	This study
***clh-1 (qa900)***	XA900	[20]
***clh-1 (qa901)***	XA901	[20]
***unc-89 (gk509355)***	VC40193	[50]
***unc-89 (gk509355)***	BLC532	This study

### *C*. *elegans* synchronization

Gravid adults were rinsed off plates using 2 ml of M9 buffer (22.1 mM KH_2_PO_4_, 42.3 mM Na_2_PO_4_, 85.6 mM NaCl) and then transferred to tubes for centrifugation at 4300 rpm for 5 minutes. Pelleted worms were resuspended in 400 μl of bleach solution (22.7% bleach, 0.1 M NaOH). When ~ 90% of the eggs were released, the reaction was stopped with 10 ml of M9 buffer. Eggs were centrifuged at 4300 rpm for 5 minutes and washed with M9 buffer twice. The pelleted eggs were then resuspended in 100 μl M9 buffer and inoculated onto seeded NGM plates.

### Quantitative real-time PCR

One day old adult hermaphrodites were rinsed off plates using 2 ml of M9 buffer and transferred into tubes containing 12 ml M9 buffer. Tubes were centrifuged for 3 minutes at 2400 rpm prior to the pellet being washed 4 times with M9. The pellet was then resuspended in 1 ml TRIzol reagent (ThermoFisher) and exposed to 6 cycles of liquid N_2_ for 30 seconds and bath of 37°C for 2 minutes. The solution was then transferred into fresh tubes and mixed with 200 μl chloroform. After 5 minutes incubation on ice, the tubes were centrifuged for 15 minutes at 15000 rpm at 4°C. The top transparent layer was then transferred to a fresh tube containing 800 μl of isopropanol and centrifuged for 10 minutes at 15000 rpm at 4°C. The pellet was then resuspended in 75% ethanol solution and centrifuged again for 5 minutes at 15000 rpm at 4°C. Finally, the pellet was resuspended in water and heated at 62°C for 10 minutes. The RNA concentration was measured using a spectrophotometer and only samples with OD_260/280_ between 1.8 and 2 were used for further analysis. One μg of RNA per sample was used for reverse transcription with the High-Capacity RNA-to-cDNA kit (Applied Biosystems) according to manufacturer’s instructions. For PCR amplification, 25 ng of cDNA were used with FAM dye labeled probes (Table 4, ThermoFisher) and TaqMan Universal Master Mix II in a CXF Connect Real-Time PCR detection system (Bio-Rad) following manufacturer’s instructions. For the *rnp-4* gene, the SYBR Green PCR method was used due to lack of commercial TaqMan *rnp-4* FAM dye labeled specific probe. Briefly, SYBR Green qPCR experiments were performed using PowerUp SYBR Green PCR Master Mix (Applied Biosystems, USA), following the manufacturer’s instructions. cDNA (25 ng) and 50 nM of the paired-primer mix were used for each reaction. The melting curve was performed and analyzed to make sure there were no nonspecific PCR products. To measure pre-mRNA levels with SYBR chemistry, extracted RNA samples were processed to remove genomic DNA before reverse transcription using a DNase kit (Qiagen, Netherlands) and following manufacturer’s instructions. The pre-RNA *clh-2* and *clh-4*, the *rnp-4* and *pmp-3* primers’ sequences used for the SYBR Green method are shown in Table 4. The gene *pmp-3* was used as an endogenous calibrator for both methods of qPCR. For the SYBR Green method, *pmp-3* primers were designed at the same location as *pmp-3* FAM dye labeled probe. The *pmp-3* primers are also in Table 4. The relative mRNA levels were calculated using the 2^-ΔΔCt^ method [51,52]. Wild type was used as the reference sample, taken as 1-fold expression level, is indicated on each figure legend.

**Table 4:** Probes and primers used for qRT-PCR amplification for TaqMan and SYBR chemistries. We note that all probes selected span exon-exon junctions that are shared between different splice variants of the same clh gene, so they do not distinguish between splice variants.

Gene	Assay ID	Probe spans exon boundary
***clh-1***	Ce02420887_g1	1–2
***clh-1***	Ce02420885_g1	15–16
***clh-2***	Ce02439134_g1	11–12
***clh-2***	Ce02439147_g1	9–10
***clh-3***	Ce02434744_g1	14–15
***clh-4***	Ce02494081_g1	14–15
***clh-4***	Ce02494095_g1	9–10
***clh-5***	Ce02436786_g1	8–9
***clh-6***	Ce02482293_g1	1–2
***unc-89***	Ce02416187_g1	27–28
***sax-3***	Ce02419838_g1	11–12
***ergo-1***	Ce02470935_m1	4–5
**eiF4AIII**	Ce02418330_g1	1–2
***smg-2***	Ce02414128_m1	3–4
***pmp-3***	Ce02485188_m1	4–5
**Primers for *rnp-4* used for SYBR chemistry**		
	**Sequence**	**Probe spans exon boundary**
**Forward**	5’- AACGCAGAAGGAAGCCAACG -3’	2–3
**Reverse**	5’- TCAGCGCTTTCCAGAAGTCT -3’	
**Primers for *clh-2* pre-mRNA used for SYBR chemistry**		
	**Sequence**	**Probe spans exon boundary**
**Forward**	5’- CCGATTCTTCCTGTTTTGGTGAGT -3’	2–3
**Reverse**	5’- TGAGATGAGGTGCGAGTTGTAGAAT -3’	
**Primers for *clh-4* pre-mRNA used for SYBR chemistry**		
	**Sequence**	**Probe spans exon boundary**
**Forward**	5’- TTGCCGGTCATGGTGAATTGATC -3’	2–3
**Reverse**	5’- ATTTGATGGTGGGATATCCGGAAGG -3’	
**Primers for *pmp-3* used for SYBR chemistry**		
	**Sequence**	**Probe spans exon boundary**
**Forward**	5’- GGAATTCTTTCGTATCTTAT -3’	4–5
**Reverse**	5’- ATTCCGTGAAACAATTCCAT -3’	

### Body measurements

To measure body length and width, we immobilized synchronized 1 day old adults in 2% agarose (in M9 buffer) pads using 100 mM sodium azide. We used a Evos FL Auto 2 Imaging System (Invitrogen) microscope to acquire images with an 40x objective (Olympus). Acquisition was done with the Evos FL Auto software. To determine the length, the distance from the tip of the nose to the end of the tail was measured in animals that were laying straight on the agar pad. The width was determined by measuring the distance from vulva opening to back of the worm. Fiji (ImageJ) was used in both cases for data analysis [53]. Measures and ratio width/length were plotted on Prism 8 for Windows (Version 8.4.2).

### Nose touch

Nose touch assays were performed as previously described [27]. In brief, healthy 1 day old adults were placed in a NGM plate containing a thin layer of OP50 and allow to crawl for 30 minutes. An eyelash was placed perpendicular to a forward moving animal so the worm would touch it with the nose while crawling forward. A response was recorded as positive if the worm showed an aversive response (reversal or head withdraw) or as negative if the worm kept moving forward over, under or along the eyelash. Each worm was tested 5 times with an interval of at least 30 seconds between touches. The average response of each worm was calculated and used for data curation (see S1 Table). The experiments were performed blind to genotype.

### Brood size

The quantification of the brood size was performed as previously described [54]. Individual worms were picked at L1 stage into separate plates containing empty OP50 or HT115 *E*. *coli* transformed with the target RNAi construct. For L4 to young adult assays, worms were picked at L4 stage. After reaching day one adulthood, worms were transferred into fresh plates for 5 consecutive days. Progenies from each plate were counted at late larva to adult stage.

### Knockdown by dsRNA feeding

A 770 bp exon-rich sequence from the genomic *rnp-4* gene was amplified by PCR using the following primers: forward (5’CTTAAGCTTAGAGATGGAGGATGTGGTGGC) and reverse (5’GTAGCTAGCTCAGCGCTTTCCAGAAGTCT). A 1128 bp exon-rich sequence from the genomic *clh-1* gene was amplified by PCR using the following primers: forward (5’GACTCAGGCTTAGGCTTAGG) and reverse (5’CTCCAACCACGGCATAAAGTCC. A 995 bp exon-rich sequence from the genomic eiF4AIII gene was amplified by PCR using the following primers: forward (5’CGTCGTAATCTTCGTACCCGAG) and reverse (5’CTCCGTTGGATAGTATTTGGGTCTTAG). The PCR products were then separately cloned into a L4440 vector containing T7 polymerase promoters to read the sequence in both sense and antisense. The vectors were transformed into HT115 *E*. *coli* that were then used to inoculate NGM plates containing IPTG. The HT115 *E*. *coli* strains expressing the L4440 vector containing a 584 bp exon-rich sequence from the genomic *mag-1* gene, a 651 bp exon-rich sequence from the genomic *smg-2* gene, a 1155 bp exon-rich sequence from the genomic *ZC144*.*5* gene, or a ≅ 1 kb exon-rich sequence from the genomic *ergo-1* gene were part of the Ahringer library [55] and were a gift from Kevin Collins. *C*. *elegans* eggs were seeded on the RNAi plates and allowed to grow for 2.5 days to adulthood prior to RNA extraction [45]. We observed reduced brood size in *rnp-4* RNAi plates, as previously reported, supporting RNAi efficiency in our hands [19], as well as almost complete sterility associated with eiF4AIII RNAi treatment.

### Statistics

For qRT-PCR, the values obtained with the 2^-ΔΔCt^ method, which avoid a false depiction of the variation, were used for statistical analysis between the target samples and their own reference sample (wild type or control) using unpaired t-test [56,57]. For phenotypic comparisons, ANOVA followed by Tukey’s was used. The statistics used for each graph are reported in the figure legends. The software Prism 8 for windows, version 8.4.2. was used.

## Supporting information

S1 Fig Schematic representation of the *clh-1* mRNA alleles, qRT-PCR with different primers, and *ergo-1* RNAi. **Related to Fig 1.** Drawings represent the spliced *clh-1* WT and mutant alleles. Exons are numbered. The position of the first introduced premature STOP codon is indicated. (B-D) mRNA levels of *clh-1*, *clh-2* and *clh-4* genes in WT and mutant alleles by qPCR using probes different from the ones used for Fig 1. Thus, a probe spanning exons 15–16 was used for *clh-1*, one spanning exons 9–10 was used for *clh-2*, and one spanning exons 9–10 was used for *clh-4*. (E-F) Pre-mRNA levels of *clh-2* and *clh-4* in WT, *ok658* and *qa901* mutants (G-J) Knockdown of *ergo-1* by RNAi feeding in WT, *ok658* and *qa901* worms. The *clh-1* mRNA levels are smaller for both mutants as compared to WT (G). The RNAi of *ergo-1* causes upregulation of *clh-2* mRNA levels (H). The *clh-4* mRNA levels are not modified under *ergo-1* RNAi treatment in both mutants (I). The *ergo-1* mRNA levels are smaller in RNAi as compared to NGM conditions (J). (B-J) *pmp-3* was used as internal control. Data are expressed as mean ± SEM and normalized to WT levels or NGM condition, that were taken as 1. Three independent experiments with three technical replicates were performed. Dashed lines indicate values from Fig 1 and are shown here for comparison: blue (panel I), corresponding to *clh-4* mRNA levels of *ok658* in NGM (2.1, from Fig 1E); red (panels H and I), corresponding to *clh-2* and *clh-4* mRNA levels, respectively, of *qa901* in NGM (0.31 from Fig 1C, and 0.47 from Fig 1E, respectively). The horizontal black dotted line corresponds to WT or control conditions, as indicated. All statistical differences are reported in the graphs (unpaired two-tailed t-test). Data used for this figure are reported in the S1 Table.
(TIF)

S2 Fig Body width and length in control and knockdown conditions, and brood size in eIF4AIII RNAi. **Related to Figs 2 and 5.** Body width and length of WT and *clh-1* mutants in control conditions and *clh-1* RNAi, (A-B), *rnp-4* RNAi (C-D), *mag-1* RNAi (E-F), and *smg-2* RNAi (G-H). Data are expressed as individual data points (open circles) and as violin plots (A-B: n = 44, 45, 27, 25, and 20, respectively; C-D: n = 19, 26, 14, and 20, respectively; E-F: n = 19, 17, 21, and 20, respectively; and G-H: n = 26, 21, 24, and 28, respectively). Statistical analysis was by one-way ANOVA followed by Tukey’s post test (*p<0.05, **p<0.01, ***p<0.001, ****p<0.0001). The horizontal dashed lines represent the values obtained in control conditions (from Fig 2C, WT: gray, *ok658*: dark blue, *qa900*: blue, *qa901*: light blue). Data used for this figure are reported in the S1 Table.
(TIF)

S3 Fig RNP-4 is needed for transcriptional adaptation. **Related to Fig 4.** (A-C) *clh-3*, *clh-5*, and *clh-6* mRNA levels, respectively, in *ok658* and *qa901* mutants treated with *rnp-4* RNAi. (D) *clh-1* mRNA levels in *ok658* and *qa901* mutants in which *rnp-4* was knocked down, showing that *clh-1* transcripts are reduced. (E) *clh-1* mRNA levels in *ok658* and *qa901* worms in which *rnp-4* was knocked down compared to their levels in control conditions, showing that mRNA degradation is reduced in *qa901* worms. (F) *rnp-4* mRNA in control conditions and *rnp-4* knockdown showing efficacy of the knockdown. (G) mRNA levels of *sax-3* in *unc-89* mutants upon knockdown of *rnp-4*, showing rescue of transcriptional adaptation. (H) *unc-89* mRNA levels in *unc-89* worms in wich *rnp-4* was knocked down, showing that *unc-89* transcript is still reduced when *rnp-4* is kncoked down. (I-K) *clh-1*, *clh-2*, and *clh-4* mRNA levels, respectively, in *ok658*, *qa900*, and *qa901* mutants in which control gene *ZC155*.*4* was knocked down. Data are expressed as mean ± SEM and normalized to WT levels or control conditions (NGM), that were taken as 1. The horizontal black dotted line corresponds to WT or control conditions, as indicated. The *pmp-3* mRNA levels were used as internal control. Three independent experiments with three technical replicates were performed. The dashed green line corresponds to mRNA levels of *sax-3* in *unc-89* worms in control conditions (from Fig 3G). All statistical differences are reported in the graphs (unpaired two-tailed t-test). See S1 Table for the data.
(TIF)

S4 Fig MAG-1 and eiF4AIII are needed for transcriptional adaptation. **Related to Fig 4.** (A) *clh-1* mRNA levels in *ok658* and *qa901* worms in which *mag-1* was knocked down, showing that *clh-1* transcripts are reduced. (B) *clh-1* mRNA levels in *ok658* and *qa901* worms in which *mag-1* was knocked down compared to their levels in control conditions, showing that mRNA degradation is reduced in *qa901* worms. (C) *mag-1* mRNA in control conditions and *mag-1* knockdown showing efficacy of the knockdown. (D) mRNA levels of *sax-3* in *unc-89* worms in *mag-1* RNAi, showing rescue of transcriptional adaptation. (E) *clh-1* mRNA levels in *ok658* and *qa901* worms in which eiF4AIII was knocked down, showing that *clh-1* transcripts are reduced. (F) *clh-1* mRNA levels in *ok658* and *qa901* worms in which eiF4AIII was knocked down compared to their levels in control conditions, showing that mRNA degradation is reduced in *qa901* worms. (G) eiF4AIII mRNA in control conditions and eiF4AIII knockdown showing efficacy of the knockdown. (H) *clh-1* mRNA levels in *ok658* and *qa901* worms in which *smg-2* has been kncoked down. (I) *clh-1* mRNA levels in *ok658* and *qa901* worms in which *smg-2* was knocked down compared to their levels in control conditions, showing that mRNA degradation is reduced in both strains. (J) *smg-2* mRNA levels in *ok658* and *qa901* mutants in control and *smg-2* RNAi, showing the efficacy of RNAi. (K) mRNA levels of *sax-3* in *unc-89* mutants upon knockdown of *smg-2*, showing rescue of transcriptional adaptation. (L) *unc-89* mRNA levels in *unc-89* worms in which *smg-2* has been knocked down. Data are expressed as mean ± SEM and normalized to WT levels or NGM conditions, that were taken as 1. The horizontal black dotted line corresponds to WT or control conditions, as indicated. The *pmp-3* mRNA levels were used as internal control. Three independent experiments with three technical replicates were performed. Dashed lines correspond to mRNA levels of *sax-3* in *unc-89* worms in control conditions (green, from Fig 3G). All statistical differences are reported in the graphs (unpaired two-tailed t-test). See S1 Table for the data.
(TIF)

S5 Fig RNP-4 and MAG-1 are not required during development for transcriptional adaptation. **Related to Fig 5.** (A-F) mRNA levels of *clh-1*, *clh-2*, and *clh-4* in *clh-1* mutants upon knockdown of *rnp-4* from L4 to Young Adult (L4toYA). (G-L) and (M-S) Same as in A-F for *mag-1* and *smg-2* RNAi, respectively. Data are expressed as mean ± SEM and normalized to WT levels or NGM condition, that were taken as 1 (black dotted line). Three independent experiments with three technical replicates were performed, and *pmp-3* mRNA levels were used as internal control. Dashed lines correspond to mRNA levels of *qa901* in control (red, from Fig 1C and 1E) and mRNA levels of *ok658* in control (blue, from Fig 1E). The horizontal black dotted line corresponds to WT or control conditions, as indicated. All statistical differences are reported in the graphs (unpaired two-tailed t-test). Data used for this figure are reported in the S1 Table.
(TIF)

S1 Table Data and statistics. File containing all the data points used for the main and supplementary figures, as well as the statistic calculations.
(XLSX)
